# Adverse Effects of Formaldehyde Vapor on Mouse Sperm Parameters and Testicular Tissue

**Published:** 2013-03-03

**Authors:** Shahram Vosoughi, Ali Khavanin, Mojdeh Salehnia, Hassan Asilian Mahabadi, Abdolhossein Shahverdi, Vahid Esmaeili

**Affiliations:** 1 Department of Occupational and Environmental Health, School of Medical Sciences, Tarbiat Modares University, Tehran, Iran; 2 Department of Anatomical Sciences, School of Medical Sciences, Tarbiat Modares University, Tehran, Iran; 3 Department of Embryology at Reproductive Biomedicine Research Center, Royan Institute for Reproductive Biomedicine, ACECR, Tehran, Iran

**Keywords:** Formaldehyde, Mouse, Sperm, Testosterone, Testis

## Abstract

**Background:**

Formaldehyde (FA), one of the simplest organic molecules, is a flammable, pungent, irritating and colorless gas. This study aimed to investigate the effects of various concentrations of FA vapor on sperm parameters and testicular tissue.

**Materials and Methods:**

In this experimental study, we randomly assigned 36 adult male mice to one control and two experimental groups (n=12 for each group). The control group (C) did not receive FA. Group F1 (low concentration) was exposed to 10 ppm FA vapor and the F2 (high concentration) group was exposed to 20 ppm FA vapor. FA was administered for ten days, eight hours per day for both groups. At the end of the exposure period, half of the animals in each group were sacrificed 24 hours after exposure to detect any short-term effects; the rest of the mice were sacrificed 35 days later to assess for long-term effects. Sperm parameters were analyzed by Computer-assisted Sperm Analyzer (CASA) and histological changes determined. In addition, we studied changes in testosterone hormone. Data were analyzed by one-way ANOVA followed by the Scheffe test using SPSS software.

**Results:**

Long-term effects of FA in the experimental groups included significant reductions in sperm cell numbers and sperm viability. A drastic reduction in progressive motility and increased abnormal sperm percentage (p<0.001) compared with the control group was also noted. Histological study of testes specimens in the experimental group revealed displacement of germinal cells, along with degeneration of Leydig cells and seminiferous tubules.

**Conclusion:**

Exposure to FA vapor can destroy testicular structure and decrease percentages of concentration, viability, normal morphology, and progressive motility, in addition to increasing the percentage of immotile sperm.

## Introduction

It is important to realize that sperm quality in humans and other animals has decreased (1) over the last 50 years, during which considerable changes have occurred in the physical, chemical, biological and socio-cultural environments of humans (2, 3).

Some recent studies have reported the effects of exposure to occupational chemicals and physical hazards on semen quality (4). The National Institute of Occupational Safety and Health (NIOSH) has introduced infertility due to occupational exposure to harmful chemical factors as a major research subject (1, 5). Because of the wide use of formaldehyde (FA, H_2_CO) in industry, science and households, the potential for its occupational or environmental exposure is increasing (6).

Industries or occupations of significant FA exposure include medical specialties (coroners, hospital housekeeping staff, and laboratory workers), embalmers, industrial (FA synthesis, molding compound, decorative laminates, plastic moldings and photographic films), textile and wood workers (plywood, particle board and furniture) (7).

Inhalation of vapors can produce irritation to the eyes, nose and upper respiratory system. Whilst occupational exposure to high FA concentrations may result in respiratory irritation and asthmatic reactions, it may also aggravate pre-existing asthma (8). Skin reactions following exposure to FA are common because the chemical can be both irritating and allergenic (9).

The harmful effects of FA in the air are welldocumented for the respiratory system, such as nasal squamous cell carcinoma and mutagenity (10). However, its effects on other systems and organs are still being studied. Chowdhury et al. (11) have noted the inhibition of steroidogenesis, disruption of Leydig cells and spermatogenesis arrest that resulted from intraperitoneal injections of FA at dosages of 5, 10 and 15 mg/kg in rats. Özen et al. (12) have shown that exposure to FA (10 and 20 ppm) caused a severe decrease in Leydig cells in mouse testes.

The negative impact of FA exposure and sperm parameters was investigated in some studies. Tang et al. (13) observed the teratogenic and cytotoxic effects of FA. In their study, administration of high intraperitoneal doses of FA caused changes in seminiferous epithelial cells.

However, reports concerning the effects of FA on sperm parameters are few and insufficient. In the present research we have investigated the effect of FA vapor on sperm parameters and testicular tissue. We used the high levels of FA vapor concentrations in the workplace (10 and 20 ppm) and studied their effects on sperm cells at two experimental time points. The effects of these vapor concentrations on mean seminiferous tubular diameters (STD) and serum testosterone levels were also investigated.

## Materials and Methods

### Formaldehyde (FA) production and treatment environment

In this experimental study, mice were placed in a vitreous Plexiglass quadrangular chamber (30×25×29 cm) that contained two holes for in and out flows of air. Air circulation in the chamber was at a fixed flow rate (12 times per hour) maintained by air pumps. The exposure chamber temperature was 22 ± 2°C. FA gas was generated by thermal depolymerization of Paraformaldehyde (Merck AG, Darmstadt, Germany) at 70-90°C according to a method described by Chang et al. (14). FA concentration in the exposure chamber reached a desirable range by changing the airflow, using a micro valve, and control by a rotameter. FA concentration was measured and monitored four times each hour by a photo ionization detector (Photocheck +5000, Ionscience Co., UK). The preciseness of the measurement was controlled according to the 3500 method recommended by National Institute of Occupational Health and Safety (NIOSH) with a sensitivity of less than 0.1% ppm (5, 15).

### Animals and treatment

This experimental study was performed on 36 normal, eight-week old male NMRI mice (25-35 g). The animals were purchased from Iran Pasteur Institute and maintained in the animal house at Tarbiat Modares University according to standard laboratory conditions in terms of temperature (22- 24ºC), light (12 hours light:12 hours dark), ventilation and free access to food and drinking water. The experiments were approved by the Institutional Animal Care and Use Committee of Tarbiat Modares University (Tehran, Iran).

The mice were randomly assigned to three groups (n=12) based on the study design. The control group (C) was maintained under experimental conditions but mice were not exposed to FA. Experimental group F1 was exposed to FA (10 ppm) eight hours per day (8:00 am- 4:00 pm) for ten consecutive days. Experimental group F2 was exposed to FA (20 ppm) eight hours per day (8:00 am- 4:00 pm) for ten consecutive days.

The population in each group (n=12) was determined based on the results from previous studies and preliminary experiments (16, 17). Other researchers reported that exposure to inhaled FA at 10 and 20 ppm which led to liver toxicity (16).

### Epididymal sperm preparation and sperm quality evaluation

For epididymal sperm preparation, mice were sacrificed by cervical dislocation either 24 hours or 35 days after the end of the exposure period. The cauda epididymides were dissected and placed in 1 ml pre-warmed Ham's F-10 (nutrient mixture- Ham-X1, Gibco, UK) culture medium with 10% fetal bovine serum (FBS, Gibco, UK). Gentle agitation along with tearing of the tissue was applied to enable spermatozoa to swim into the medium in a Falcon culture dish (18,19). Semen samples were incubated at 37°C for 30 minutes before analysis of sperm parameters.

Sperm count and motility were analyzed with a Computer-assisted Sperm Analyzer (CASA) as described by Krause (18). The CASA system consisted of a phase contrast microscope (Eclipse E-200, Nikon Co., Japan) with a heat plate equipped with Sperm Class Analyzer® software (SCA, full research version 5.1, Microptic Co., Barcelona, Spain). Images were captured by a video camera (Basler Vision, A312FC at 50 fps, Tecnologie Co., Ahrensburg, Germany) at 100x magnification. For this purpose, 4 µl sperm samples were placed in a standard count analysis chamber (Leja, Nieuw Vennep Co., Netherlands). The loaded chamber was placed on the warm plate of the microscope (37°C) for 3 minutes before analysis. Then, specimens were observed with a Nikon microscope 10x/0.25 negative phase contrast field Ph1 BM, with an intermediate magnification of 0.7 and a green filter. Several fields of view were captured and at least 400 spermatozoa counted for each analysis.

performed eosin-Y staining by mixing 10 µl of the sperm sample with 10 µl of dye (0.5% wt/vol, Merck Chemical Co., Germany) on a microscope slide which was then covered with a coverslip. A total of 200 sperm cells were counted within a few minutes after the addition of the dye (19). Evaluation of live (unstained) and dead (red stained) spermatozoa was performed with a phase contrast microscope at x400 magnification.

For the assessment percentage of the normal sperm morphology, we used SpermBlue® fixative and stain. Briefly, once the smear air dried at room temperature it was fixed for 10 minutes by placing the slide with the dried smear in a staining tray that contained SpermBlue® fixative. The fixed smear was stained for 10 minutes by immersing the slide in a staining tray that contained SpermBlue® stain (20). Morphologic assessment was performed at x1000 magnification with a phase contrast microscope, and at least 200 spermatozoa were counted on each slide.

### Histological analysis and morphometric technique

For histological examination, testicular tissues were dissected and samples fixed in Bouin’s fixative for 24 hours, then processed by a graded ethanol series and embedded in paraffin. The paraffin sections were cut in 5 µm thick slices and stained with hematoxylin and eosin for light microscopic examination (21, 22).

Sections were viewed and photographed by a microscope (Magnum-3, Ceti, England) with an attached camera (Sony-DSC-H50). We compared the integrity of seminiferous tubules and interstitial cells, including Leydig cells, with the control group. An estimate of STD was performed by examining 20 fields in 5 histological sections from each testis (22), using digitalized microscopic images (x10) with the software Image Tools 3.0 (Obtain from http://compdent.uthscsa.edu/dig/itdesc.html).

### Testosterone hormone measurement

Serum was separated by centrifugation at 3000×g for 15 minutes, then collected and stored at -20°C until analysis. Serum testosterone level was evaluated following the standard protocol supplied by a kit (Monobind Inc., USA, Product code: 3775-300) and read by a fully automated ELISA reader (23).

### Statistical analysis

All statistical analyses were performed using SPSS statistical software version 11.5 (SPSS, Inc., Chicago, IL). All data were expressed as mean ± SD. Comparisons between control and FA-exposed groups were performed using one-way analysis of variance (ANOVA), followed by the Scheffe test. The level of statistic significance was set at p<0.05.

## Results

### Formaldehyde concentration

The mean FA concentrations in the exposure chamber were 10.89 ± 0.76 ppm for group F1 and 19.79 ± 1.56 ppm for group F2. During all exposure sessions we registered minor, insignificant fluctuations in the FA vapor concentration.

### Testicular weight

**Fig 1:**
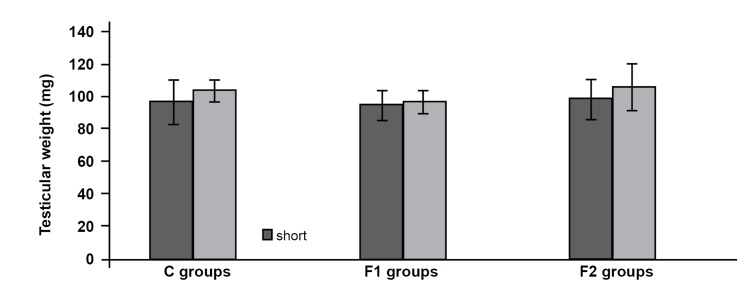
Testicular weight in mice sacrificed 24 hours (short-term analysis) and 35 days (long-term analysis) after exposure.

The mean of testes weights in experimental groups F1 and F2 were 94.72 ± 8.88 and 98.58 ± 12.34 (mgs), respectively during short-term analysis. The mean testes weights in experimental groups F1 and F2 were 96.85 ± 7.15 and 106.03 ± 14.77(mgs), respectively during long-term analysis. No statistical decrease was observed in all experimental groups compared to the control group (p<0.05) in mice sacrificed at 24 hours and 35 days after exposure (Fig 1).

### Effects of formaldehyde on sperm parameters

Sperm parameters in mice assessed 24 hours after FA exposure are shown in table 1. We observed no significant changes in sperm count, motility and percentage of normal morphology among the experimental groups compared to the control group (p>0.05) for short-term analysis (Table 1). FA exposure at 20 ppm was associated with a decreased percentage of progressive motility. Also in this concentration there was a significant decrease (p<0.05) in percentage of sperm viability compared with the control group (Table 1).

Sperm parameters in mice sacrificed 35 days after FA exposure are summarized in table 1. The progressive motility of sperm cells in both experimental groups compared to the control group decreased significantly (p<0.001). Regarding sperm count and normal morphology, a significant decrease was observed in all exposure groups compared to the control group (p<0.001) in long-term analysis. A comparison of exposure groups with one another showed no statistical difference (p>0.05) in terms of these two parameters (Table 1).

**Table 1:** Differences in mouse sperm parameters in experimental groups at 24 hours (short-term analysis) and 35 days (longterm analysis) after exposure (mean ± SD)


Experimental group	Sperm count (10^6^)	Progressive motility (%)	Non-progressive motility (%)	Sperm viability (%)	Normal morphology (%)

**Short-term analysis**						
**CS**	4.77 ± 0.56	42.87 ± 4.05	37.62 ± 7.09	19.52 ± 4.05	82.17 ± 3.49	81.33 ± 2.73
**F1S**	3.93 ± 1.09	39.72 ± 5.72	34.37 ± 4.94	26.08 ± 6.12	75.33 ± 5.89	75.67 ± 3.08
**F2S**	3.73 ± 0.53	35.22 ± 3.63	34.42 ± 5.56	30.35 ± 4.32*^b^	70.88 ± 4.92*^b^	75.33 ± 3.14
**Long-term analysis**						
**CL**	5.08 ± 0.65	44.47 ± 2.88	37.93 ± 3.12	17.58 ± 5.34	83.33 ± 4.63	82.17 ± 2.23
**FIL**	2.87 ±0.51**^a^	26.65 ±1.61**^a^	33.08 ± 5.61*^a^	40.27 ± 6.38**^a^	61.33 ± 5.85**^a^	71.67 ± 2.73**^a^
**F2L**	2.58 ±0.44**^b^	24.17±3.81**^b^	28.57 ± 4.56	47.28 ± 6.68**^b^	54.67 ± 5.96**^b^	68.83 ± 2.79**^b^


*; p<0.05, **; p<0.001, C; Control groups. F1; Exposed to formaldehyde (FA) vapor (10 ppm), F2; Exposed to FA vapor (20 ppm), S; Short-term analysis (24 hours), L; Long- term analysis (35 days) a; Difference between experimental group (F1) and control group, b; Difference between experimental group (F2) and control group and c; Difference between experimental groups.

**Fig 2:**
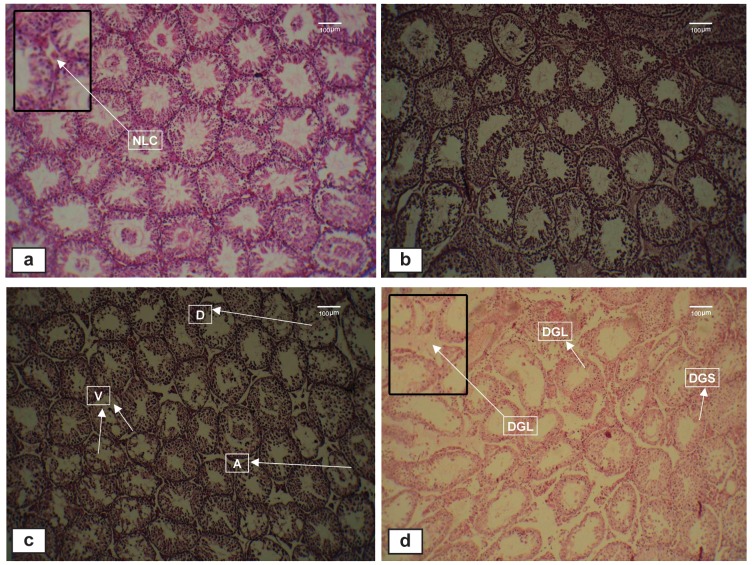
Light micrographs of testicular sections stained with hematoxylin and eosin (x 100). a. Normal histological structure of seminiferous tubules in the control (C) group. b. Exposure to formaldehyde (FA) vapor at 10 ppm (short-term analysis) showing normal spermatogenic cells and seminiferous tubules, similar to the control group. c. Exposure to FA vapor at 10 ppm (long-term analysis) showing atrophy and disruption of germ cells in seminiferous tubules and vacuolization of seminiferous epithelium. d. Exposure to FA vapor at 20 ppm (long-term analysis) showing degeneration in Leydig cells and seminiferous tubules. A; Atrophy, D; Disruption, DGL; Degeneration in Leydig cells, DGS; Degeneration in seminiferous tubules, NLC; Normal Leydig cell and V; Vacuolization.

### Testicular histopathology

Histological examination of the testis showed numerous structural changes in the experimental groups (long-term analysis) compared to the control group. The main pathological changes included seminiferous tubule atrophy, increase in the spaces between germ cells, degeneration of Leydig cells, disintegration of seminiferous epithelial cells and degeneration of a number of seminiferous tubules. No histological changes were seen in the control specimens (Fig 2 a-d).

**Table 2:** Comparison of the means of seminiferous tubular diameter (STD) in control and exposure groups (one-way ANOVA and Scheffe Test)


Groups*	STD in mice sacrificed 24 hours after exposure (mean ± SD)	P value	STD in mice sacrificed 35 days after exposure (mean ± SD)	P value

**C**	233.33 ± 6.055		235.17 ± 5.742	
**F1**	218.83 ± 5.913	0.281	210.33 ± 5.465	0.007
**F2**	216.50 ± 7.503	0.147	203.67 ± 5.989	0.001


Group C; Control, group F1; Exposed to formaldehyde (FA; 10 ppm) and group F2; Exposed to FA (20 ppm). P values; vs. control group.

**Table 3:** Comparison of the means of serum testosterone concentration in control and exposure groups (one-way ANOVA and Scheffe Test)


Groups*	STD in mice sacrificed 24 hours after exposure (mean ± SD)	P value	STD in mice sacrificed 35 days after exposure (mean ± SD)	P value

**C**	3.82 ± 0.345		3.62 ± 0.153	
**F1**	2.58 ± 0.159	0.000	3.24 ± 0.227	0.134
**F2**	1.96 ± 0.176	0.000	3.08 ± 0.253	0.009


Group C; Control, group F1; Exposed to formaldehyde (FA, 10 ppm) and group F2; Exposed to FA (20 ppm). P values; vs. control group.

The morphometric findings indicated that the mean STD decreased in both short and long-term analysis in the experimental groups compared with the control groups,but it was significant (p<0.05) only in long-term analysis (Table 2).

### Effects of formaldehyde on serum testosterone level

Short-term analysis of testosterone levels showed a significant difference (p<0.001) between the F1 (2.58 ± 0.16 ng/ml) and F2 (1.95 ± 0.18 ng/ml) groups compared to the control (3.82 ± 0.35 ng/ ml). The comparison between the F1 and F2 groups was statistically significant (p<0.05; Table 3).

## Discussion

The results of the present study indicated that sperm count, viability, progressive motility and normal morphology in all exposure groups significantly reduced in long-term analysis compared with the control group.

These results involve novel information that deals with the adverse effects of FA on sperm progressive motility in two specified time points, one day after exposure and 35 days after exposure. Regeneration of the full cycle of epithelium cells in seminiferous tubules in mice is 8.6 days, whereas for spermatogenesis, the period is 35 days. Thus changes in sperm physiological parameters are observed better after a period of 35 days (24, 25).

With respect to the findings of our study, subacute exposure in the workplaces and high concentrations of FA vapor exposure can lead to histological changes to the seminiferous tubules and Leydig cells. These structural changes are related to the time of analysis.

There is a possibility that FA will cross the blood testis barrier and induce oxidative stress and lipid peroxidation by increasing reactive oxygen species (ROS). Thus, in our study degeneration of Leydig cells and decreased seminiferous tubule diameters have likely resulted from oxidative damage from FA vapor. Degeneration of Leydig cells is possibly in charge of decreased testosterone levels which affect sperm parameters of sperm progressive motility, count and normal morphology.

In this study, the results obtained from analysis of the effects of FA on testis weight have shown no statistically significant difference between experimental groups and control group both in shortterm and long-term analyses. The non-significant differences possibly were the result of individual differences in mice. Some researchers have reported the decrease in testis weight due to the oral use of FA (5 mg/kg) in quail (26) and FA inhalation (10 mg/m^3^) during two weeks of exposure in rats (27). Zahra et al. (28) studied mice that were administered FA (5 and 10 mg/kg) for 40 days. At the end of the exposure period, there were no significant differences observed between the experimental and control groups, which supported the findings of the present study in terms of testis weight.

In the current study, animals sacrificed 24 hours after FA exposure did not show histopathological changes in testes tissues, in contrast, disintegration of seminiferous epithelial cell, increasing the distances between the seminiferous tubules and decreasing the mean of STD observed in long-term analysis (35 days after the end of exposure).

The increase in inter-tubular specimens appears to parallel the decrease in STD, however the number of Leydig or interstitial cells in FA-exposed groups during in long-term analysis decreased when compared with the control group. We demonstrated that the testosterone level improved 35 days after the end of the exposure; however it was significantly lower than either the control group or level of testosterone measured at 24 hours after exposure.

The increase in inter-tubular specimens appears to parallel the decrease in STD, however the number of Leydig or interstitial cells in FA-exposed groups during in long-term analysis decreased when compared with the control group. We demonstrated that the testosterone level improved 35 days after the end of the exposure; however it was significantly lower than either the control group or level of testosterone measured at 24 hours after exposure.

Ozen et al. (12) revealed that sub-chronic exposure to FA (5-10 ppm) for 91 days caused significant reductions in tubular diameters. The experimental study by Golalipour et al. (29) also showed that exposure to FA vapor for 18 weeks in the rat induced histological changes in seminiferous tubules and decreased the mean of STD.

The result of Zhou et al. (27) stated that exposure to FA vapor (10 mg/m^3^ for two weeks) led to seminiferous tubule atrophy, a decrease in spermatogenesis cells and disintegration of seminiferous epithelial cells.

In the current study, FA vapor exposure at the concentration and duration mentioned caused histological changes in the seminiferous epithelium and decreased the mean STD in mice. The morphometric findings obtained in the present study, in a way, were consistent with the findings of Golalipour et al. (29).

Kose et al. (30) studied the effect of FA on the rat reproductive system. In their study, experimental animals were exposed to FA vapor (10 ppm/1 hour) for 35 days. They observed the detrimental effects of FA on sperm count, motility and normal morphology. These researchers reported a relationship between reduction of STD and decreased numbers of Leydig cells in rats. Henkel et al. (31) determined a direct correlation between sperm motility and decrease in Leydig cells.

According to the study by Tang et al. (13), intraperitoneal injections of FA at doses of 0.2, 2, and 20 (mg/kg) negatively impacted sperm count, viability and sperm motility in rats. They reported that seminiferous tubule atrophy and degeneration of seminiferous tubules lead to reduced sperm counts.

The findings of long-term sperm analysis in our study were compatible with the results of the above studies. Further, the results of the long-term analysis (35 days after the end of exposure) revealed a significant difference between experimental groups F1, F2 and the control group in terms of progressive motile sperm.

The present research showed that FA vapor decreased progressive motile sperm. According to Mazzilli et al. (32) non-progressive, immotile and abnormal sperm can produce anion super oxidase, which is an oxidative factor by itself that can decrease sperm quality, including sperm motility.

Decreasing sperm motility depends on various factors for which the reason is still unknown. Increasing the production of ROS and attenuation of testis tissue neurogenesis are two factors that decrease sperm motility; the latter plays a more pivotal role in creating dysfunctions to sperm morphology (33, 34).

## Conclusion

Our study showed that FA vapor adversely affects mice sperm parameters, including decreased counts, viability, normal morphology, progressive motility and increased percentages of immotile sperm. It has been recommended that more attention should be paid to the relationship between the FA vapor and gene expression in male fertility.
